# Preoperative Cobb angle-guided surgical decision-making between percutaneous kyphoplasty and short-segment fixation with vertebroplasty in stage III Kümmell disease without neurological deficits: a two-center retrospective study

**DOI:** 10.3389/fendo.2026.1887883

**Published:** 2026-07-17

**Authors:** Jun Hu, Long Tang, Yi Liu, Hong Yang, Yu Qiao, Xin Wang

**Affiliations:** 1 Department of Spine Surgery, Wuhan Fourth Hospital, Hubei Provincial Sports Medicine Center, Hubei Provincial Clinical Research Center for Orthopaedics, Hubei Key Laboratory of Sports Injury and Precision Therapy, Wuhan, China; 2 Department of Spine Surgery, Suining Central Hospital, Suining, China; 3 Department of Orthopaedics and Sports Orthopaedics, School of Medicine and Health, TUM University Hospital, Technical University of Munich, Munich, Germany

**Keywords:** Cobb angle, Kümmell disease, neurologically intact, percutaneous kyphoplasty, risk stratification, short-segment fixation

## Abstract

**Objective:**

To compare the clinical and radiographic outcomes of percutaneous kyphoplasty (PKP) and short-segment fixation with vertebroplasty (SSF+VP) in patients with stage III Kümmell disease (KD) without neurological deficits, and to evaluate whether the preoperative local Cobb angle can guide risk stratification for surgical decision-making.

**Methods:**

This two-center retrospective study included 117 patients with stage III KD without neurological deficits who underwent surgery between January 2019 and December 2024. Among them, 71 underwent PKP and 46 underwent SSF+VP. Perioperative parameters, VAS, ODI, local Cobb angle, Cobb angle loss, cement distribution, and cement leakage were compared. In this exploratory analysis, residual mild pain was defined as a final follow-up VAS ≥2. In the PKP group, the association between Cobb angle loss and final follow-up VAS was analyzed. ROC analysis was performed to assess the predictive value of the preoperative Cobb angle for residual mild pain after PKP. Patients were further stratified according to the optimal preoperative Cobb angle cut-off, and 2,000 bootstrap resamples were used for internal validation.

**Results:**

Baseline characteristics were comparable. PKP was associated with shorter operative time, less blood loss, smaller cement volume, shorter hospital stay, and greater early VAS and ODI improvement than SSF+VP. However, at final follow-up, SSF+VP showed lower VAS, while ODI was comparable. Radiographically, SSF+VP achieved lower postoperative and final Cobb angles, less Cobb angle loss, more diffuse cement distribution, and less cement leakage. In the PKP group, Cobb angle loss correlated positively with final VAS (rho=0.542, *p* < 0.001). The preoperative Cobb angle predicted residual pain after PKP, with an optimal cut-off of 25.95°. After stratification, final VAS did not differ between procedures in the mild-moderate kyphosis subgroup, whereas SSF+VP showed better intermediate-term pain outcomes in the severe kyphosis subgroup. Bootstrap validation supported the stability of this risk-stratified finding.

**Conclusion:**

Both PKP and SSF+VP improved pain and function in stage III KD without neurological deficits. PKP enabled faster early recovery, whereas SSF+VP provided better correction maintenance and intermediate-term pain outcomes in patients with severe preoperative kyphosis. Preoperative Cobb angle-based stratification may help guide individualized surgical selection.

## 1 Introduction

Kümmell disease (KD) is a delayed ischemic osteonecrotic disorder of the vertebral body that predominantly occurs in elderly patients with osteoporosis. Its typical clinical course is characterized by a transient asymptomatic period after minor trauma, followed by progressive persistent back pain, vertebral collapse, and local kyphotic deformity (1, 2). With population aging and the increasing incidence of osteoporotic vertebral compression fractures, KD has become increasingly relevant in clinical spine surgery (3). Based on clinical and radiographic manifestations, KD is commonly classified into different stages. Stage III KD is usually characterized by marked vertebral collapse, local kyphotic deformity, and formation of an intravertebral vacuum cleft (IVC), and some patients may present with radiographic evidence of spinal canal compromise or spinal cord compression (4). For patients with stage III KD who have not yet developed neurological deficits, timely reconstruction of spinal stability, pain relief, and prevention of long-term deformity progression before the onset of neurological impairment represent key clinical challenges.

The optimal surgical strategy for stage III KD without neurological deficits remains controversial. Percutaneous kyphoplasty (PKP) has been widely used in the treatment of osteoporotic vertebral compression fractures and selected patients with KD because of its minimal invasiveness, short operative time, limited blood loss, and rapid early recovery. Through balloon expansion and bone cement augmentation, PKP can partially restore vertebral height, enhance the stability of the affected vertebra, and provide rapid pain relief (5). However, patients with stage III KD often present with more severe vertebral structural destruction and local kyphotic deformity, and whether cement augmentation alone can maintain correction over the long term remains uncertain (6). In some patients, Cobb angle loss, recurrence of kyphotic deformity, cement leakage, or residual pain during long-term follow-up may occur after PKP, which may limit the applicability of PKP in patients with severe kyphosis (7–9).

Short-segment fixation with vertebroplasty (SSF+VP) provides an alternative treatment option for stage III KD. This procedure reconstructs local stability through a posterior short-segment pedicle screw system and augments the affected vertebra with bone cement, thereby achieving kyphosis correction, vertebral reinforcement, and segmental stabilization simultaneously (10, 11). Compared with PKP alone, SSF+VP may theoretically be more advantageous for maintaining local sagittal alignment and reducing the risk of long-term loss of kyphosis correction. However, this procedure is associated with greater surgical trauma, longer operative time, and increased intraoperative blood loss, which may impose a higher perioperative burden on elderly patients with osteoporosis (11). Therefore, clinical decision-making for stage III KD without neurological deficits should not simply involve choosing between a “minimally invasive” procedure and “instrumented fixation”; rather, it requires a balanced consideration of early recovery, surgical trauma, long-term stability, and pain relief.

Previous studies comparing PKP and SSF+VP for KD have mainly focused on their overall clinical efficacy, showing that both procedures can improve pain and functional disability (12). However, clear and quantifiable preoperative criteria are still lacking for identifying which patients are more suitable for PKP and which patients may benefit more from SSF+VP. In fact, the degree of preoperative kyphosis varies substantially among patients with stage III KD. Kyphotic deformity may, to some extent, reflect the severity of vertebral collapse and local sagittal imbalance, and may also indicate a higher risk of difficulty in maintaining correction after cement augmentation alone. For patients with mild preoperative kyphosis, PKP may be sufficient to achieve pain relief and stability reconstruction. In contrast, for patients with more severe preoperative kyphosis, cement augmentation alone may have limited ability to withstand local mechanical stress over the long term, whereas posterior fixation combined with vertebral augmentation may be more favorable for long-term pain outcomes (13). Therefore, identifying a simple, objective, and preoperatively available radiographic indicator is of important clinical significance for guiding individualized surgical decision-making.

The local Cobb angle is one of the most commonly used and intuitive radiographic parameters for evaluating the severity of kyphotic deformity in KD (14). Unlike postoperative Cobb angle loss, the preoperative local Cobb angle can be directly measured on routine preoperative radiographs, making it highly practical in clinical decision-making. If the preoperative Cobb angle can predict residual mild pain after PKP, it may further serve as a useful tool for preoperative risk stratification and help surgeons make a more reasonable choice between PKP and SSF+VP. However, evidence remains limited regarding whether the preoperative Cobb angle can predict residual mild pain after PKP in patients with stage III KD without neurological deficits, and whether it can be used to guide surgical decision-making.

Based on this background, the present study retrospectively included 117 patients with stage III KD without neurological deficits who underwent PKP or SSF+VP at two centers. First, we compared the differences between the two procedures in terms of perioperative parameters, pain relief, functional improvement, maintenance of Cobb angle correction, bone cement distribution, and cement leakage. Subsequently, focusing on the PKP group, we analyzed the relationship between Cobb angle loss and the degree of pain at the final follow-up, and further evaluated the predictive value of the preoperative local Cobb angle for residual pain after PKP. Finally, all patients were stratified according to the optimal cut-off value of the preoperative Cobb angle, and intermediate-term pain outcomes after PKP and SSF+VP were compared across different degrees of kyphosis. Bootstrap internal validation was performed to assess the stability of this stratification strategy. This study aimed to propose a simple risk stratification method based on the preoperative Cobb angle, thereby providing evidence to support individualized surgical decision-making for patients with stage III KD without neurological deficits.

## 2 Materials and methods

### 2.1 Patient selection and grouping

This retrospective study included 117 patients with stage III KD without neurological deficits who underwent surgical treatment at Wuhan Fourth Hospital and Suining Central Hospital between January 2019 and December 2024. All patients were diagnosed with stage III KD based on clinical symptoms and radiographic examinations, including plain radiography, computed tomography (CT), and magnetic resonance imaging (MRI). According to the surgical procedure performed, the patients were divided into the PKP group (n = 71) and the SSF+VP group (n = 46). The choice of surgical approach was not randomized. When the evaluation indicated that bone cement augmentation alone was considered sufficient to relieve pain and achieve local vertebral stability, the PKP procedure is given priority. SSF+VP was more commonly considered in patients with more severe vertebral collapse, greater local kyphotic deformity, suspected local instability, or a greater need for correction and maintenance of sagittal alignment, provided that the patient’s general condition allowed a more invasive procedure. The final treatment decision was made by senior spine surgeons at both hospitals after comprehensively considering imaging findings, surgical tolerance, and comorbidities, and following discussions with the patient and their family.

The inclusion criteria were as follows: (1) a confirmed diagnosis of stage III KD based on clinical symptoms and radiographic examinations; (2) preoperative imaging findings showing marked vertebral collapse and intravertebral cleft formation, with or without spinal canal compromise; (3) no definite preoperative neurological deficits; and (4) complete clinical, radiographic, and follow-up data.

The exclusion criteria were as follows: (1) comminuted bilateral pedicle fractures; (2) pathological fractures caused by tumors, tuberculosis, or other non-osteoporotic etiologies; (3) preoperative neurological impairment; (4) severe medical comorbidities that precluded surgical tolerance; and (5) incomplete follow-up data.

This study was approved by the institutional ethics committees of the participating hospitals (No. KY2024-204-01; KYLLKS20260016), and the requirement for informed consent was waived due to the retrospective nature of the study.

### 2.2 Surgical procedures

**Figure 1:**
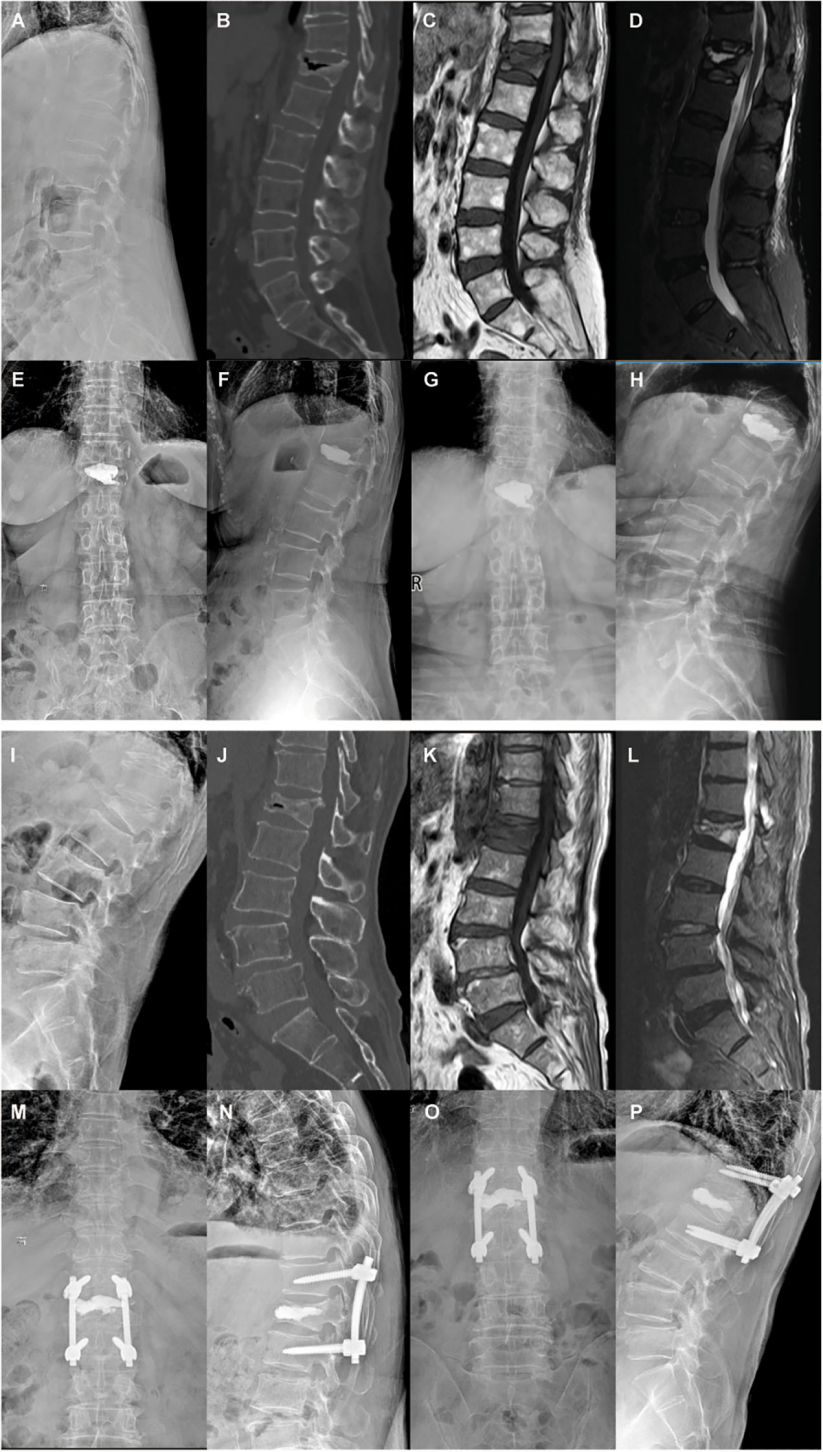
Representative radiographic images of stage III Kümmell disease (KD) without neurological deficits treated with percutaneous kyphoplasty (PKP) or short-segment fixation with vertebroplasty (SSF+VP). **(A–H)** A representative patient treated with PKP. **(A–D)** Preoperative lateral radiograph, sagittal CT, and sagittal MRI show marked vertebral collapse, intravertebral cleft (IVC) formation, and local kyphotic deformity. **(E, F)** Postoperative anteroposterior and lateral radiographs showing bone cement augmentation after PKP. **(G, H)** Final follow-up anteroposterior and lateral radiographs showing the cement distribution and vertebral alignment after PKP. **(I–P)** A representative patient treated with SSF+VP. **(I–L)** Preoperative lateral radiograph, sagittal CT, and sagittal MRI show severe vertebral collapse, IVC formation, and local kyphotic deformity. **(M, N)** Postoperative anteroposterior and lateral radiographs showing posterior short-segment fixation combined with vertebroplasty. **(O, P)** Final follow-up anteroposterior and lateral radiographs showing maintained vertebral alignment, fixation status, and cement distribution.

All procedures at each center were performed by the same team of senior spine surgeons. Patients in the PKP group were placed in a prone hyperextension position. Under C-arm fluoroscopic guidance, bilateral transpedicular puncture was performed to access the affected vertebra. After satisfactory puncture placement was confirmed, balloons were inserted and gradually inflated to restore vertebral height and improve the local kyphotic deformity. Subsequently, bone cement injection was strictly controlled under continuous fluoroscopic monitoring. If early signs of cement migration toward high-risk anatomical areas were detected, injections were immediately stopped. In vertebrae with cortical disruption or posterior wall defects on preoperative imaging, gelatin sponge packing was used before cement injection to reduce potential leakage pathways. The procedure was completed once satisfactory cement distribution was achieved and no obvious risk of cement leakage was observed. Patients in the SSF+VP group were placed in the prone position under general anesthesia. The affected vertebra and adjacent segments were exposed through a posterior midline incision. Four universal pedicle screws were implanted into the adjacent vertebrae above and below the affected vertebral body. These screws engage seamlessly with pre-bent titanium rods and minimize the risk of screw channel loosening within fragile, osteoporotic bone during active traction and reduction. After connection with pre-bent titanium rods, distraction and reduction were performed through the screw–rod system to correct the local kyphotic deformity and restore vertebral alignment. After satisfactory reduction was achieved, vertebroplasty of the affected vertebra was performed under C-arm fluoroscopic guidance, and bone cement was injected into the affected vertebra to enhance vertebral stability. Representative preoperative, postoperative, and final follow-up radiographic images of patients treated with PKP and SSF+VP are shown in **Figure 1**.

### 2.3 Postoperative management

Postoperative management was generally similar between the two groups. If the patient’s general condition permitted, they were encouraged to begin gradual mobilization while wearing a thoracolumbar brace. Typically, the brace was worn during early postoperative mobilization and daily activities. In addition, all patients were advised to receive systemic treatment for osteoporosis, including routine calcium and vitamin D supplementation. At the same time, based on factors such as the patient’s BMD, fracture risk, underlying medical conditions, and financial situation, individualized anti-osteoporotic treatment, such as bisphosphonates, denosumab, or teriparatide, was prescribed when clinically indicated.

### 2.4 Radiographic assessment

All radiographic parameters were independently measured by two physicians who were not involved in the surgical procedures, and the mean of the two measurements was used for statistical analysis. The assessed parameters included the local Cobb angle on radiographs obtained preoperatively, 3 days postoperatively, and at the final follow-up, Cobb angle loss, and cement distribution pattern. The local Cobb angles measured 3 days postoperatively and at the final follow-up were used to evaluate postoperative kyphosis correction and maintenance of correction. Inter-observer reliability was assessed using intraclass correlation coefficients (ICC) with 95% confidence intervals based on a two-way mixed-effects model for absolute agreement. Cobb angle loss was defined as the difference between the local Cobb angle at the final follow-up and that at 3 days postoperatively, reflecting the degree of correction loss and maintenance of kyphotic deformity after surgery. The cement distribution pattern was assessed on radiographs obtained 3 days postoperatively and classified as either diffuse or mass. The diffuse pattern was defined as good interdigitation between the cement and surrounding trabecular bone, with relatively dispersed cement distribution within the vertebral body. The mass pattern was defined as cement mainly confined to the intravertebral cleft region, presenting as an isolated mass-like distribution.

### 2.5 Clinical evaluation and outcome definitions

Perioperative data included operative time, intraoperative blood loss, length of hospital stay, and procedure-related complications. Complications mainly included cement leakage, adjacent vertebral fracture, instrumentation-related complications, and other perioperative adverse events. Clinical outcomes were evaluated using the visual analogue scale (VAS) for pain intensity and the Oswestry Disability Index (ODI) for back-related functional status. VAS and ODI were recorded preoperatively, 3 days postoperatively, and at the final follow-up to compare early recovery and intermediate-term clinical outcomes between the two surgical procedures.

Because the overall pain level at the final follow-up was relatively low and the median final follow-up VAS in the PKP group was 2, a final follow-up VAS score of ≥2 was used as a study-specific operational definition of residual mild pain in this exploratory analysis. This threshold was intended to distinguish patients who still had perceptible mild pain from those who were nearly pain-free, rather than to define surgical failure, poor pain control, or moderate-to-severe residual pain. According to standard clinical pain metric validations, a VAS score in the range of 1 to 4 typically reflects mild discomfort rather than severe functional impairment (15). Therefore, this binary endpoint was used only to explore whether preoperative radiographic severity could identify patients at higher risk of residual mild pain after PKP.

### 2.6 Correlation analysis of Cobb angle loss, ROC-based threshold analysis, and subgroup validation

In the PKP group, the relationship between Cobb angle loss and pain intensity at the final follow-up was further analyzed to determine whether insufficient maintenance of kyphosis correction after PKP was associated with residual mild pain. Spearman correlation analysis was performed to assess the association between Cobb angle loss and final follow-up VAS. This analysis was intended to explore the relationship between loss of structural correction and residual pain outcomes.

After confirming the association between Cobb angle loss and final follow-up pain intensity, ROC curve analysis was further performed to evaluate the discriminative ability of Cobb angle loss for residual mild pain. A final follow-up VAS of ≥2 was used as the binary outcome variable, and Cobb angle loss was used as the predictor. The optimal cut-off value was determined according to the Youden index. This analysis was used to identify the threshold of structural correction loss associated with residual mild pain.

Subsequently, to determine whether preoperative radiographic parameters could predict the risk of residual mild pain after PKP, ROC curve analysis was performed in the PKP group. A final follow-up VAS of ≥2 was used as the binary outcome variable, and the preoperative local Cobb angle was used as the predictor. The optimal cut-off value was determined according to the Youden index. This analysis aimed to clarify whether the severity of preoperative kyphotic deformity could serve as a predictor of residual mild pain after PKP.

Based on the cut-off value derived from ROC analysis of the preoperative Cobb angle, all patients, including those in the PKP and SSF+VP groups, were further stratified according to the optimal preoperative Cobb angle threshold. The mild-to-moderate kyphosis subgroup was defined as a preoperative Cobb angle of <25.95°, whereas the severe kyphosis subgroup was defined as a preoperative Cobb angle of ≥25.95°. Within each Cobb angle stratum, the final follow-up VAS was compared between PKP and SSF+VP to evaluate differences in intermediate-term pain outcomes between the two procedures across different degrees of preoperative kyphotic deformity. This subgroup analysis was used to determine whether the preoperative Cobb angle threshold had potential reference value for surgical decision-making.

### 2.7 Bootstrap internal validation

To validate the stability of the subgroup comparison results after stratification based on the preoperative Cobb angle, bootstrap internal validation was performed. Using a preoperative Cobb angle of 25.95° as the stratification threshold, all patients were divided into the mild-moderate kyphosis subgroup and the severe kyphosis subgroup. During the bootstrap procedure, stratified resampling with replacement was performed to preserve the original subgroup structure, and the resampling process was repeated 2,000 times. After each resampling iteration, the final follow-up VAS was compared between PKP and SSF+VP within the mild-moderate and severe kyphosis subgroups, respectively, and the mean VAS difference of SSF+VP relative to PKP was calculated. The bootstrap results were expressed as the resampled mean difference, 95% confidence interval, median *p* value, and proportion of resampling iterations with *p* < 0.05, which were used to evaluate the robustness of the subgroup comparison results.

In addition, bootstrap ROC analysis was further performed to assess the stability of the preoperative Cobb angle cut-off value. After each bootstrap resampling iteration, ROC curves were reconstructed using residual mild pain as the binary dependent variable and the preoperative Cobb angle as the predictor. The corresponding optimal cut-off value, AUC, sensitivity, and specificity were extracted based on the Youden index. This analysis was used to evaluate the stability of the preoperative Cobb angle risk threshold during the resampling process.

### 2.8 Statistical analysis

All statistical analyses were performed using R software. Continuous variables were first tested for normality. Normally distributed continuous variables are presented as mean ± standard deviation and were compared between groups using the independent-samples t-test. Non-normally distributed continuous variables are presented as median and interquartile range and were compared using the Wilcoxon rank-sum test. Categorical variables are presented as numbers and percentages and were compared using the χ² test or Fisher’s exact test, as appropriate. Correlation analysis was performed using Spearman correlation analysis, and the correlation strength and statistical significance were expressed as the correlation coefficient rho and P value. ROC curves were used to evaluate the discriminative ability of Cobb angle loss and the preoperative Cobb angle for residual mild pain. The AUC, 95% confidence interval, sensitivity, and specificity were calculated. The optimal cut-off value was determined according to the Youden index. Bootstrap internal validation was performed using stratified resampling with replacement, with 2,000 resampling iterations. Resampling was stratified according to “Cobb angle stratum × surgical procedure” to preserve the original subgroup structure. Bootstrap results were expressed as the resampled mean difference, standard error, percentile-based 95% confidence interval, median P value, and proportion of resampling iterations with *p* < 0.05. In addition, bootstrap ROC analysis was performed to assess the stability of the preoperative Cobb angle cut-off value for predicting residual mild pain. After each resampling iteration, the ROC curve was recalculated, and the AUC, Youden index-derived cut-off value, sensitivity, and specificity were recorded. All statistical tests were two-sided, and *p* < 0.05 was considered statistically significant.

## 3 Results

### 3.1 Comparison of baseline characteristics and perioperative parameters between the two groups

Follow-up duration was 11–19 months in the PKP group and 12–17 months in the SSF+VP group. All patients included in the final analysis had complete clinical and radiographic follow-up data, whereas patients with incomplete follow-up data were excluded according to the exclusion criteria. Ultimately, a total of 117 patients with stage III KD without neurological deficits were included in this study, including 71 patients in the PKP group and 46 patients in the SSF+VP group. No significant differences were observed between the two groups in terms of sex distribution, age, bone mineral density, or follow-up duration, indicating that the preoperative baseline characteristics were comparable between the two groups (*p*>0.05, **Table 1**).

**Table 1:** Comparison of baseline characteristics between the two groups.

Variable	PKP	SSF+VP	*p* value
Case	71	46	
Gender			
Male	22	15	1.000
Female	49	31	
Age	70.75 ± 6.29	71.13 ± 5.83	0.611
BMD (T value)	-3.12 ± 0.35	-3.04 ± 0.32	0.174
Follow-up (months, range)	14.24 ± 1.89 (11–19)	13.83 ± 1.48 (12-17)	0.265
Operation time (min)	33.87 ± 8.13	113.74 ± 17.38	**<0.001**
Estimated blood loss (ml)	23.52 ± 7.76	167.85 ± 46.74	**<0.001**
Bone cement volume (ml)	3.56 ± 1.14	5.67 ± 0.95	**<0.001**
Length of hospital stay (d)	4.85 ± 1.29	9.80 ± 2.54	**<0.001**

Bolded values indicate statistical significance (p<0.05).

Regarding perioperative parameters, the PKP group showed significantly lower operative time, intraoperative blood loss, cement volume, and length of hospital stay than the SSF+VP group (**Table 1**). The operative time in the PKP group was 33.87 ± 8.13 min, which was significantly shorter than that in the SSF+VP group (113.74 ± 17.38 min; *p* < 0.001). Intraoperative blood loss was also significantly lower in the PKP group than in the SSF+VP group (23.52 ± 7.76 ml vs. 167.85 ± 46.74 ml; *p* < 0.001). Regarding cement volume, the PKP group received 3.56 ± 1.14 ml, whereas the SSF+VP group received 5.67 ± 0.95 ml, indicating a greater cement volume in the SSF+VP group (*p* < 0.001). In addition, the length of hospital stay was significantly shorter in the PKP group than in the SSF+VP group (4.85 ± 1.29 d vs. 9.80 ± 2.54 d; *p* < 0.001). These findings indicate that the two groups had generally comparable preoperative baseline characteristics. Compared with SSF+VP, PKP demonstrated perioperative advantages, including shorter operative time, less intraoperative blood loss, and shorter hospital stay, reflecting its less invasive nature and faster early recovery.

### 3.2 Changes in pain and functional outcomes

There was no significant difference in preoperative VAS between the two groups. The preoperative VAS was 7.00 (6.00, 8.00) in the PKP group and 7.00 (6.00, 8.00) in the SSF+VP group (*p*>0.05), indicating comparable baseline pain intensity between the two groups. After surgery, VAS decreased significantly in both groups compared with preoperative values, suggesting that both procedures effectively relieved pain in patients with stage III KD without neurological deficits. At 3 days postoperatively, the VAS was significantly lower in the PKP group than in the SSF+VP group [3.00 (2.00, 4.00) vs. 4.00 (3.00, 4.75); *p* < 0.001], indicating that PKP provided better early postoperative pain relief. At the final follow-up, VAS further improved in both groups compared with the values at 3 days postoperatively. The final follow-up VAS was 2.00 (1.50, 2.00) in the PKP group and 1.50 (1.00, 2.00) in the SSF+VP group, with a significantly lower value in the SSF+VP group (*p* < 0.01). These findings suggest that although PKP provided superior early pain relief, SSF+VP was associated with more favorable intermediate-term pain outcomes.

Regarding ODI, no significant difference was observed between the two groups preoperatively. The preoperative ODI was 73.00 (69.00, 76.00) in the PKP group and 72.00 (69.00, 75.00) in the SSF+VP group (*p*>0.05), indicating comparable baseline functional disability. At 3 days postoperatively, ODI decreased significantly in both groups compared with the preoperative values, suggesting that both procedures improved back-related functional status. The ODI at 3 days postoperatively was significantly lower in the PKP group than in the SSF+VP group [42.00 (35.00, 48.00) vs. 50.50 (46.00, 56.50); *p* < 0.001], indicating faster early functional recovery after PKP. At the final follow-up, ODI further decreased in both groups compared with the values at 3 days postoperatively. The final follow-up ODI was 25.00 (21.00, 32.00) in the PKP group and 26.00 (21.25, 34.00) in the SSF+VP group, with no significant difference between the two groups (*p*>0.05). Overall, both procedures significantly improved pain and functional disability. PKP showed advantages in early postoperative pain relief and functional recovery, whereas SSF+VP showed more favorable pain outcomes at the final follow-up. However, intermediate-term functional recovery was comparable between the two groups (**Table 2**).

**Table 2:** Improvements in VAS score and ODI score.

	PKP	SSF+VP	*p* value
VAS scores			
Preoperative	7.00 (6.00, 8.00)	7.00 (6.00, 8.00)	0.307
Postoperative	3.00 (2.00, 4.00)^a^	4.00 (3.00, 4.75)^a^	**<0.001**
Final follow-up	2.00 (1.50, 2.00)^a,b^	1.50 (1.00, 2.00)^a,b^	**0.003**
ODI scores (%)			
Preoperative	73.00 (69.00, 76.00)	72.00 (69.00, 75.00)	0.433
Postoperative	42.00 (35.00, 48.00)^a^	50.50 (46.00, 56.50)^a^	**<0.001**
Final follow-up	25.00 (21.00, 32.00)^a,b^	26.00 (21.25, 34.00)^a,b^	0.471

^a^p < 0.001(comparison with preoperative); ^b^p < 0.001 (comparison with postoperative). Bolded values indicate statistical significance (p<0.05).

### 3.3 Comparison of radiographic parameters between the two groups

Before comparing radiographic outcomes, the inter-observer reliability of local Cobb angle measurements was assessed. The ICCs were 0.866 (95% CI, 0.812–0.905) for the preoperative Cobb angle, 0.883 (95% CI, 0.836–0.917) for the 3-day postoperative Cobb angle, and 0.906 (95% CI, 0.867–0.934) for the final follow-up Cobb angle, indicating good inter-observer reliability. There was no significant difference in the preoperative local Cobb angle between the two groups. The preoperative local Cobb angle was 28.30° (25.45°, 31.70°) in the PKP group and 27.15° (25.50°, 28.80°) in the SSF+VP group (*p*>0.05), indicating comparable baseline kyphotic deformity between the two groups. After surgery, the local Cobb angle improved significantly in both groups compared with the preoperative values, suggesting that both PKP and SSF+VP could partially correct the local kyphotic deformity. At 3 days postoperatively, the local Cobb angle was 19.70° (18.25°, 22.40°) in the PKP group and 13.60° (10.70°, 16.00°) in the SSF+VP group, with a significantly lower value in the SSF+VP group (*p* < 0.001). This finding indicates that SSF+VP provided greater immediate correction of kyphosis than PKP. At the final follow-up, the local Cobb angle was 22.50° (19.90°, 24.40°) in the PKP group and 14.75° (12.17°, 17.88°) in the SSF+VP group. Although some loss of correction was observed in both groups compared with the immediate postoperative values, the final follow-up local Cobb angle remained significantly lower in the SSF+VP group than in the PKP group (*p* < 0.001). Regarding Cobb angle loss, the value was significantly higher in the PKP group than in the SSF+VP group [2.20° (1.45°, 3.45°) vs. 0.90° (0.50°, 1.40°); *p* < 0.001]. This result suggests that, compared with SSF+VP, PKP was associated with a greater tendency toward loss of kyphosis correction after surgery, which may be related to the limited ability of cement augmentation alone to maintain vertebral height and local sagittal stability over time.

The cement distribution pattern differed significantly between the two groups (*p<*0.05). In the PKP group, 23 patients (32.4%) showed a mass pattern, and 48 patients (67.6%) showed a diffuse pattern. In the SSF+VP group, 6 patients (13.0%) showed a mass pattern, and 40 patients (87.0%) showed a diffuse pattern. The proportion of diffuse patterns was higher in the SSF+VP group, suggesting more sufficient cement dispersion within the vertebral body, which may contribute to improved overall support of the affected vertebra.

Regarding cement leakage, leakage occurred in 22 patients (31.0%) in the PKP group and 5 patients (10.9%) in the SSF+VP group, with a significantly higher leakage rate in the PKP group (*p<*0.05). Apart from bone cement leakage, the incidence of other surgery-related complications was low and varied in type, with no statistically significant differences observed. 4 patients (5.63%) in the PKP group and 2 patients (4.35%) in the SSF+VP group experienced adjacent vertebral fractures (*p*>0.05). In the SSF+VP group, no patients experienced complications related to internal fixation. No other perioperative adverse events were observed. This finding suggests that although PKP has a minimally invasive advantage, it may be associated with a relatively higher risk of cement leakage. In contrast, SSF+VP, which combines vertebral augmentation with reduction and fixation, may be more favorable for controlling cement injection pressure and distribution morphology. Overall, although the two groups had similar preoperative kyphotic deformity, SSF+VP showed advantages over PKP in immediate Cobb angle correction, maintenance of correction at the final follow-up, reduction of Cobb angle loss, and improvement of cement distribution, while also showing a lower incidence of cement leakage (**Table 3**).

**Table 3:** Comparison of radiological evaluation results.

	PKP	SSF+VP	*p* value
Cobb angle (°)			
Preoperative	28.30 (25.45, 31.70)	27.15 (25.50, 28.80)	0.089
Postoperative	19.70 (18.25, 22.40)^a^	13.60 (10.70, 16.00)^a^	**<0.001**
Final follow-up	22.50 (19.90, 24.40)^a,b^	14.75 (12.17, 17.88)^a,b^	**<0.001**
Loss of Cobb angle (°)	2.20 (1.45, 3.45)	0.90 (0.50, 1.40)	**<0.001**
Types of Bone Cement			**0.018**
Mass type	23 (32.4%)	6 (13.0%)	
Diffuse type	48 (67.6%)	40 (87.0%)	
Bone cement leakage	22 (31.0%)	5 (10.9%)	**0.012**

^a^p < 0.001(comparison with preoperative); ^b^p < 0.001 (comparison with postoperative). Bolded values indicate statistical significance (p<0.05).

### 3.4 Relationship between Cobb angle loss and residual mild pain in the PKP group

**Figure 2:**
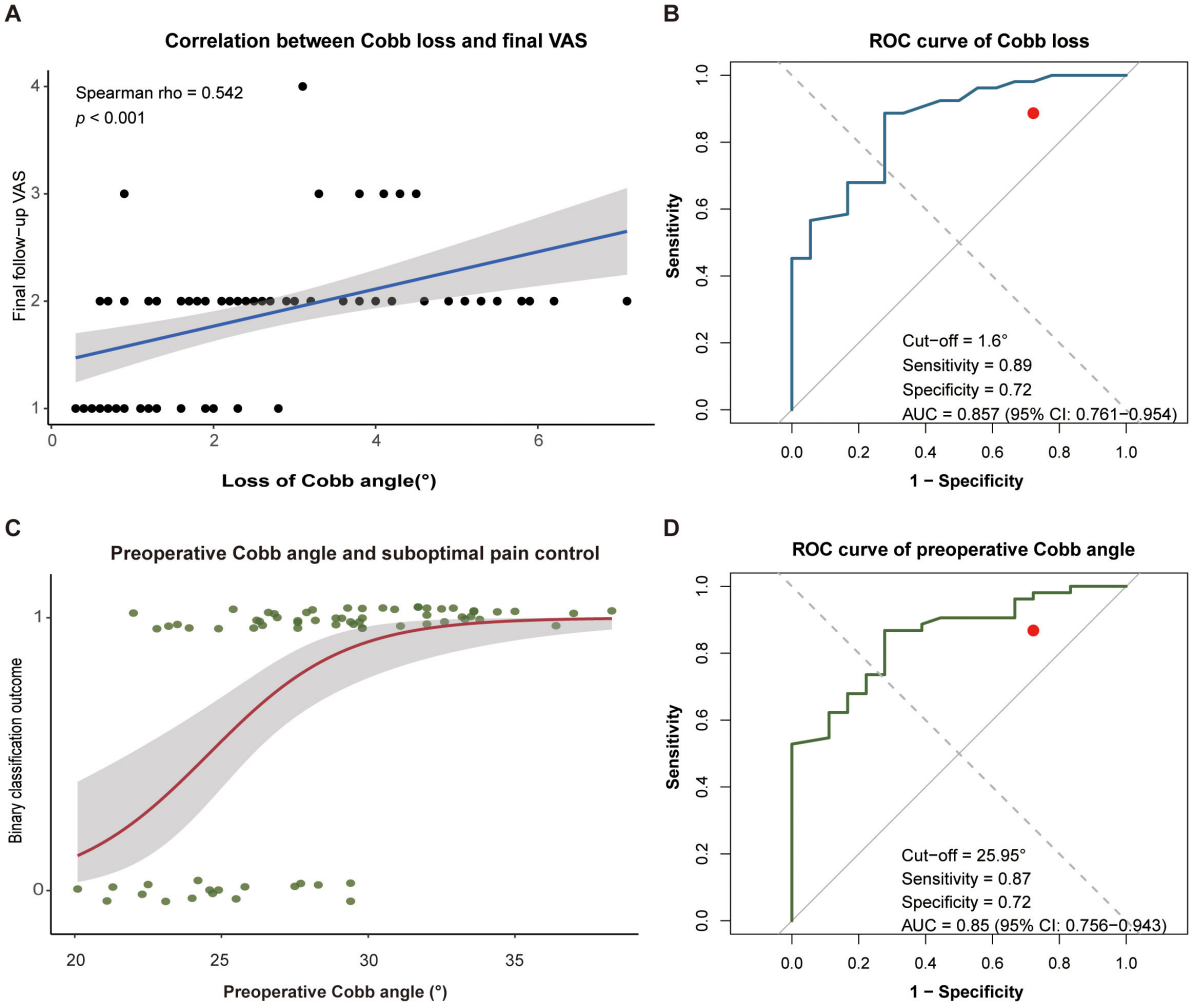
Association of Cobb angle loss and preoperative Cobb angle with residual mild pain in the percutaneous kyphoplasty (PKP) group. **(A)** Correlation analysis between Cobb angle loss and VAS at the final follow-up; **(B)** ROC curve for Cobb angle loss as a predictor of residual mild pain; **(C)** Relationship between preoperative Cobb angle and residual mild pain; **(D)** ROC curve for preoperative Cobb angle as a predictor of residual mild pain. Residual mild pain was defined as a VAS score ≥2 at the final follow-up. Red dots indicate the optimal cut-off values corresponding to the Youden index.

Given the relatively higher final follow-up VAS and more pronounced Cobb angle loss in the PKP group, we further analyzed the relationships between Cobb angle loss, pain intensity at the final follow-up, and residual mild pain within the PKP group. Spearman correlation analysis showed that Cobb angle loss was significantly positively correlated with final follow-up VAS, with a correlation coefficient of rho = 0.542 (*p* < 0.001; **Figure 2**).

ROC analysis was then performed using residual mild pain as the binary endpoint to evaluate the discriminative ability of Cobb angle loss. The results showed that Cobb angle loss had good discriminative performance for residual mild pain, with an AUC of 0.857 (95% CI, 0.761–0.954). Based on the Youden index, the optimal cut-off value of Cobb angle loss was 1.6°, with a sensitivity of 0.89 and a specificity of 0.72 (**Figure 2**). We further analyzed the relationship between the preoperative Cobb angle and residual mild pain. The scatter plot and fitted curve showed that the probability of residual mild pain increased with increasing preoperative Cobb angle (**Figure 2**). ROC analysis further demonstrated that the preoperative Cobb angle also had good predictive value for residual mild pain, with an AUC of 0.850 (95% CI, 0.756–0.943). Based on the Youden index, the optimal cut-off value of the preoperative Cobb angle was 25.95°, with a sensitivity of 0.87 and a specificity of 0.72 (**Figure 2**).

### 3.5 Subgroup comparison based on the preoperative Cobb angle cut-off value

Based on the ROC analysis of the preoperative Cobb angle for predicting residual mild pain in the PKP group, all patients were stratified according to the optimal preoperative Cobb angle cut-off value. Patients with a preoperative Cobb angle of <25.95° were defined as the mild-moderate kyphosis subgroup, whereas those with a preoperative Cobb angle of ≥25.95° were defined as the severe kyphosis subgroup. The final follow-up VAS was then compared between PKP and SSF+VP within each subgroup.

In the mild-moderate kyphosis subgroup, there were 20 patients in the PKP group and 18 patients in the SSF+VP group. The final follow-up VAS was 1.00 (1.00, 2.00) with a mean of 1.45 ± 0.69 in the PKP group and 2.00 (1.00, 2.00) with a mean of 1.39 ± 0.78 in the SSF+VP group. The mean difference calculated as SSF+VP minus PKP was -0.06, and no significant difference was observed between the two groups(*p*>0.05). In the severe kyphosis subgroup, there were 51 patients in the PKP group and 28 patients in the SSF+VP group. The final follow-up VAS was 2.00 (2.00, 2.00) with a mean of 2.02 ± 0.51 in the PKP group and 1.00 (1.00, 2.00) with a mean of 1.46 ± 0.74 in the SSF+VP group. The mean difference was -0.56, indicating a lower mean final follow-up VAS in the SSF+VP group (*p* < 0.001, **Table 4**). These findings suggest that, after stratification by the preoperative Cobb angle, the difference in intermediate-term pain outcomes between the two procedures was mainly observed in patients with severe kyphosis.

**Table 4:** Comparison of final follow-up VAS scores between PKP and SSF+VP after stratification by the preoperative Cobb angle cut-off.

Kyphosis subgroup	Group	N	Final VAS, median [IQR]	Final VAS, mean ± SD	Mean difference	*p* value
Mild-moderate kyphosis	PKP	20	1.00 (1.00, 2.00)	1.45 ± 0.69		0.822
	SSF+VP	18	2.00 (1.00, 2.00)	1.39 ± 0.78	-0.06	
Severe kyphosis	PKP	51	2.00 (2.00, 2.00)	2.02 ± 0.51		
	SSF+VP	28	1.00 (1.00, 2.00)	1.46 ± 0.74	-0.56	**<0.001**

IQR, interquartile range; SD, standard deviation.

Since the VAS is non-normally distributed, the median [IQR] was used. The mean difference was calculated based on the mean using the formula SSF+VP - PKP, therefore, the mean ± SD deviation is also provided.

*The p values were calculated using the Wilcoxon rank-sum test. Bolded values indicate statistical significance (p<0.05).

### 3.6 Bootstrap internal validation

**Figure 3:**
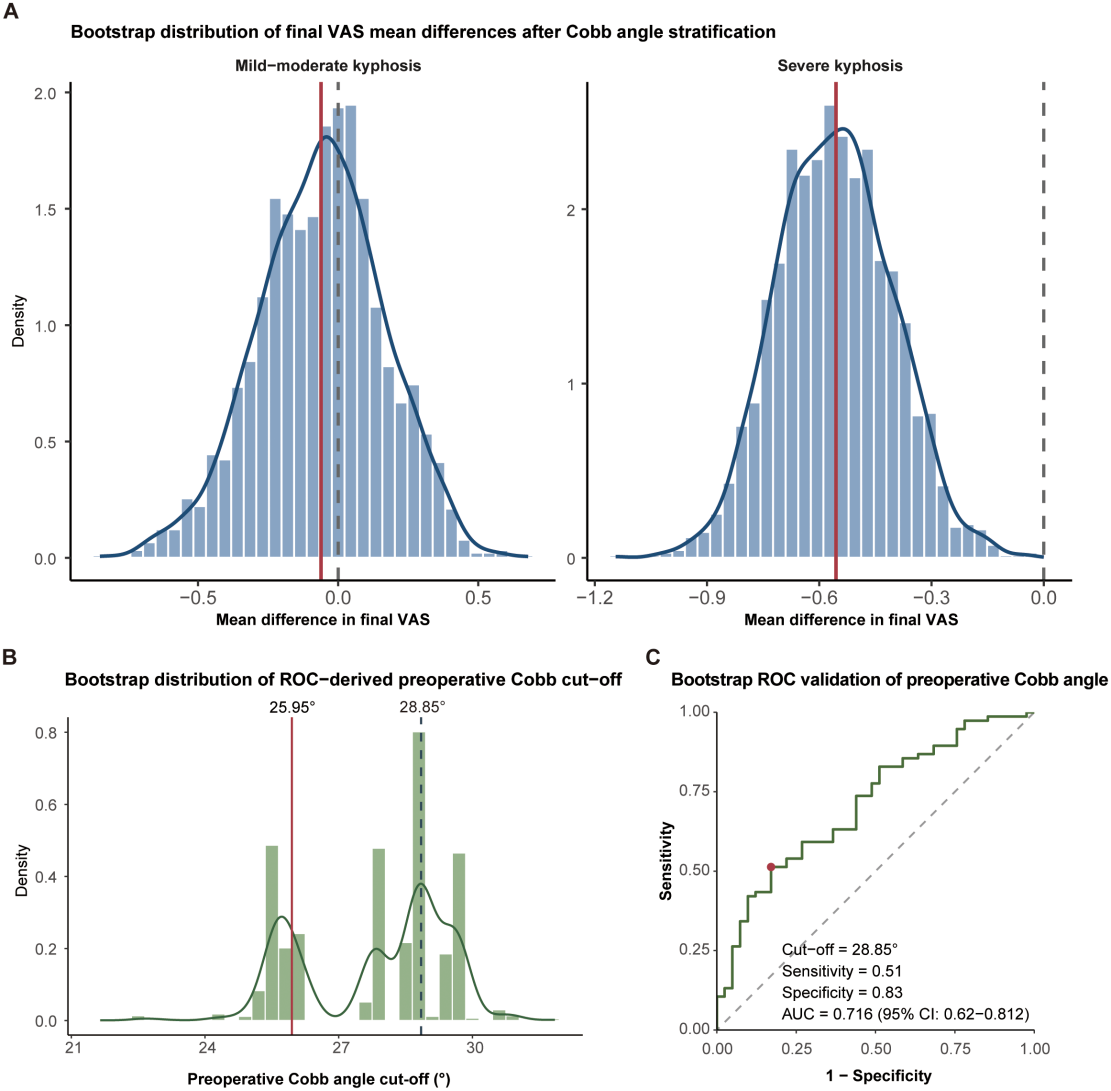
Bootstrap internal validation of subgroup comparisons and preoperative Cobb angle cut-off. **(A)** Bootstrap distributions of the mean difference in final follow-up VAS between short-segment fixation with vertebroplasty (SSF+VP) and percutaneous kyphoplasty (PKP) after stratification by the preoperative Cobb angle cut-off. The mean difference was calculated as SSF+VP minus PKP. The red solid line indicates the original estimate, and the grey dashed line indicates zero difference. **(B)** Bootstrap distribution of ROC-derived preoperative Cobb angle cut-offs. The red solid line indicates the main analysis cut-off of 25.95°, and the blue dashed line indicates the bootstrap median cut-off of 28.85°. **(C)** Bootstrap ROC validation of the preoperative Cobb angle for predicting residual mild pain.

In the mild-moderate kyphosis subgroup, the original sample showed a mean difference in final follow-up VAS of −0.06 for SSF+VP relative to PKP. After bootstrap resampling, the mean difference was −0.061, with a 95% CI of −0.53 to 0.37. The median *p* value was 0.482, and the proportion of resampling iterations with *p* < 0.05 was 6.9%. These results indicate that, among patients with mild-to-moderate kyphosis, the difference in final follow-up VAS between the two procedures was not stable during resampling, and most resampling iterations did not reach statistical significance (**Figure 3**). In the severe kyphosis subgroup, the original sample showed a mean difference in final follow-up VAS of −0.56 for SSF+VP relative to PKP. After bootstrap resampling, the mean difference was −0.558, with a 95% CI of −0.84 to −0.26. The median *p* value was <0.001, and the proportion of resampling iterations with *p* < 0.05 was 96.1%. The bootstrap distribution of the mean difference was mainly below zero, indicating that the lower final follow-up VAS observed with SSF+VP compared with PKP in the severe kyphosis subgroup remained stable during resampling (**Figure 3**; **Table 5**).

**Table 5:** Bootstrap internal validation of final follow-up VAS comparisons after stratification based on the preoperative Cobb angle cut-off.

Kyphosis subgroup	Original mean difference	Bootstrap mean difference	Bootstrap 95% CI	Median bootstrap *p* value	Proportion of *p* < 0.05
Mild-moderate kyphosis	-0.06	-0.061	-0.53 to 0.37	0.482	0.069
Severe kyphosis	-0.56	-0.558	-0.84 to -0.26	**<0.001**	0.961

CI, confidence interval. Mean difference was calculated as SSF+VP minus PKP. A negative value indicates a lower final follow-up VAS score in the SSF+VP group. Bootstrap resampling was repeated 2000 times. The bootstrap 95% CI was percentile-based. The median bootstrap p value represents the median of the p values obtained from the Wilcoxon rank-sum test across bootstrap resamples. The proportion of bootstrap iterations with p < 0.05 indicates the frequency with which the between-group difference reached statistical significance during resampling. Bolded value indicate statistical significance (p<0.05).

Bootstrap ROC analysis was further performed to assess the stability of the preoperative Cobb angle cut-off value. The ROC-derived cut-off values obtained from bootstrap resampling were mainly distributed around and to the right of the main analysis cut-off value of 25.95°, with a median cut-off value of 28.85° (**Figure 3**). In the bootstrap ROC validation, the preoperative Cobb angle showed an AUC of 0.716 (95% CI, 0.620–0.812) for predicting residual mild pain, with a corresponding cut-off value of 28.85°, sensitivity of 0.51, and specificity of 0.83 (**Figure 3**). These results indicate that the cut-off value for the preoperative Cobb angle remained within a similar risk range during resampling.

Overall, bootstrap internal validation further supported that, among patients with mild-moderate kyphosis, the difference in final follow-up pain outcomes between PKP and SSF+VP was not statistically significant. In contrast, among patients with severe kyphosis, SSF+VP showed a stable and statistically significant advantage over PKP in final follow-up pain outcomes. In addition, bootstrap ROC analysis showed that the preoperative Cobb angle cut-off value remained within a similar risk range during resampling, supporting the stability of the preoperative Cobb angle as a stratification indicator.

## 4 Discussion

The pathological feature of stage III KD is intravertebral pseudarthrosis secondary to osteoporotic vertebral fracture, and its major clinical challenges include mechanical instability, progressive spinal deformity, and the potential risk of neural compression (16, 17). Therefore, effective restoration of both immediate and sustained spinal stability is required. At present, both PKP and SSF+VP are effective treatment options for stage III KD with severe vertebral collapse but without neurological deficits, and no clear consensus has yet been established regarding the optimal surgical strategy (18, 19).

This study focused on surgical decision-making between PKP and SSF+VP for stage III KD without neurological deficits and proposed a risk stratification strategy based on the preoperative local Cobb angle. Previous studies have generally suggested that both PKP and SSF+VP can improve pain and functional disability in patients with KD, although the two procedures differ in their relative advantages regarding minimal invasiveness, maintenance of kyphosis correction, and long-term stability (11, 18, 20). A systematic review by Lu et al. also showed that both PKP and SSF+VP are generally safe and effective for KD without neurological deficits, while PKP has advantages in shortening operative time and reducing intraoperative blood loss (21). Consistent with these findings, our study showed that operative time, intraoperative blood loss, and length of hospital stay were significantly lower in the PKP group than in the SSF+VP group, and that early postoperative improvements in VAS and ODI were more pronounced after PKP. These results further support the advantage of PKP as a minimally invasive procedure in facilitating early recovery.

However, the therapeutic challenge in stage III KD does not lie solely in short-term pain relief, but rather in maintaining durable long-term stability in the context of vertebral osteonecrosis, osteoporosis, and local kyphotic deformity (16, 22). Previous studies have indicated that whether stage III KD is suitable for percutaneous vertebral augmentation alone remains controversial. The main concerns include posterior wall disruption, insufficient vertebral stability, and whether bone cement can provide sufficiently durable mechanical support (23, 24). In our study, although PKP provided faster early postoperative pain relief, the SSF+VP group showed a lower VAS at the final follow-up. In addition, the postoperative and final follow-up Cobb angles were both significantly lower in the SSF+VP group than in the PKP group, and Cobb angle loss was also smaller. These findings suggest that, for stage III KD without neurological deficits, focusing only on early postoperative outcomes may underestimate the influence of maintaining sagittal alignment over the medium to long term on pain prognosis. Therefore, after observing both a relatively higher final follow-up VAS and more pronounced Cobb angle loss in the PKP group, we considered it necessary to further clarify whether these two findings were correlated.

Further analysis showed that Cobb angle loss was significantly positively correlated with final follow-up VAS in the PKP group, indicating that insufficient maintenance of kyphosis correction was closely associated with residual mild pain. Previous studies have suggested that an important source of pain in KD is related to micromotion at the vertebral fracture site and local instability, and that the key therapeutic goal is to eliminate micromotion and reconstruct vertebral stability. Bone cement can provide immediate stability through filling and interlocking mechanisms; however, in the context of osteonecrosis, sclerosis, and bone resorption in KD, anchorage between the cement and surrounding trabecular bone may be insufficient (21, 25–28). Therefore, when further Cobb angle loss occurs after PKP, its clinical significance is not limited to a radiographic angular change; rather, it may reflect insufficient local structural support and inadequate maintenance of stability, thereby explaining why some patients continue to experience residual pain during intermediate follow-up. This is consistent with previous literature, which indicates that even after vertebroplasty, slight misalignment of sagittal plane parameters or localized microinstability may lead to long-term mechanical fatigue and significant residual back pain in elderly patients (29, 30).

In addition, our study found that the SSF+VP group showed more stable outcomes in terms of maintenance of kyphosis correction, control of Cobb angle loss, and final follow-up pain relief. This advantage may be attributable to the composite stabilization mechanism of SSF+VP. Posterior short-segment fixation can provide additional three-column mechanical support and help restore and maintain local sagittal alignment. In this study, the routine application of universal pedicle screws in the SSF+VP group provided key clinical advantages in the context of severe osteoporosis. Compared with unidirectional screws, which generate rigid toggling leverage that can easily loosen or strip the screw track during distraction, polyaxial screws help dissipate stress at the bone-screw interface. Furthermore, the multi-axial head allows for a more secure mechanical lock with contoured, pre-bent titanium rods without necessitating forceful anatomical displacement, thereby minimizing the risks of intraoperative pedicle blowout and construct failure (31, 32). Meanwhile, cement augmentation can enhance screw–bone interface stability and reduce the risk of screw loosening and correction loss in osteoporotic vertebrae. When combined with vertebroplasty of the affected vertebra, SSF+VP may provide complementary support through posterior fixation and anterior column reinforcement, thereby reducing the risks of local stress concentration and recurrent kyphosis associated with cement filling alone (33, 34). Consistent with this mechanism, our study also found a higher proportion of diffuse cement distribution and a lower rate of cement leakage in the SSF+VP group, which further supports this interpretation from both radiographic and safety perspectives.

Notably, previous studies have not reached fully consistent conclusions regarding the long-term outcomes of PKP and SSF+VP. A systematic review by Lu et al. showed no significant differences between the two procedures in final VAS, ODI, Cobb angle, or cement leakage; however, that study also emphasized that the existing evidence was mainly derived from retrospective studies with limited sample sizes and substantial patient heterogeneity (21). This suggests that comparisons based solely on the overall population may obscure the true differences among patients with different radiographic risk profiles. Our study confirmed that postoperative Cobb angle loss was associated with final pain outcomes. Therefore, we further sought to determine whether a preoperative radiographic parameter could help guide surgical decision-making. Unlike postoperative Cobb angle loss, the preoperative Cobb angle can be directly obtained from routine preoperative radiographs, is simple to measure, and is therefore readily applicable in clinical practice.

Our study found that the preoperative Cobb angle could predict residual mild pain, with an AUC of 0.850. In the PKP group, the optimal cut-off value of the preoperative Cobb angle determined by the Youden index was 25.95°. After all patients were stratified based on this threshold, no significant difference in final follow-up VAS was observed between PKP and SSF+VP among patients with mild-to-moderate kyphosis, whereas SSF+VP was significantly superior to PKP among patients with severe kyphosis. These findings indicate that surgical decision-making should not be based simply on the general assumption that “PKP is minimally invasive” or that “SSF+VP is more stable”; instead, individualized assessment should be performed according to the severity of preoperative kyphotic deformity. Similarly, Shen et al., in their comparison of percutaneous vertebroplasty and percutaneous short-segment fixation for KD, reported that both procedures were effective, but suggested that percutaneous short-segment fixation should be preferentially considered in patients with severe kyphotic deformity. This conclusion is highly consistent with the surgical selection tendency identified in the present study based on the Cobb angle threshold (35). Bootstrap internal validation further strengthened the reliability of our stratified findings. In the mild-moderate kyphosis subgroup, the bootstrap distribution of the mean difference crossed zero, suggesting that the difference in intermediate-term pain outcomes between the two procedures was not stable. In contrast, in the severe kyphosis subgroup, the mean VAS difference of SSF+VP relative to PKP remained consistently negative during resampling, and most resampling iterations reached statistical significance. More importantly, the median preoperative Cobb angle cut-off value obtained from bootstrap ROC validation was 28.85°, which was within a similar risk range to the 25.95° threshold derived from ROC analysis in the PKP group. These findings suggest that a preoperative Cobb angle greater than approximately 26°–29° may indicate a higher likelihood of residual mild pain after PKP.

From the perspective of clinical decision-making, the findings of this study support a more refined treatment strategy. For patients with mild-to-moderate kyphosis and a relatively low preoperative Cobb angle, PKP can achieve intermediate-term pain outcomes comparable to those of SSF+VP while offering less surgical trauma, shorter operative time, and faster early recovery; therefore, it still has clear clinical value. However, for patients with severe kyphosis and a preoperative Cobb angle reaching or exceeding approximately 26°–29°, PKP alone may still provide early pain relief, but it may also be associated with a higher risk of insufficient maintenance of kyphosis correction and residual mild pain. If the patient’s general condition permits, SSF+VP may represent a more reasonable option. In recent years, studies have also attempted to improve cement filling quality and reduce the risks of leakage and displacement by modifying PKP instruments or using cement-containing devices. This indicates that the treatment concept in this field is shifting from simply considering whether a procedure is “minimally invasive” toward whether stable structural support can be established (8).

KD is secondary to severe osteoporosis; therefore, surgery alone cannot fully address the underlying systemic skeletal fragility. Standard treatment for osteoporotic vertebral fractures typically includes pain management, anti-osteoporosis medications, exercise or rehabilitation, and the use of braces for protection when appropriate (36). Treatment of systemic osteoporosis is crucial for improving bone strength and reducing the risk of subsequent vertebral fractures. Studies have shown that anabolic therapies, such as teriparatide, can reduce subsequent vertebral fractures in patients with osteoporosis following vertebroplasty and improve pain-related outcomes (37, 38). Furthermore, early postoperative use of a brace can provide temporary external support and pain relief, although existing evidence suggests that its effects on radiographic parameters and long-term function remain inconsistent (39). In this study, both groups followed broadly similar postoperative management protocols, including brace protection and anti-osteoporotic therapy. Nevertheless, as this was a retrospective study, individual differences in medication adherence, brace compliance, and long-term osteoporosis control could not be fully quantified; these factors may have contributed to residual confounding.

However, this study still has several limitations. First, this was a dual-center retrospective study, and the choice of surgical procedure was not randomized. Treatment selection may have been influenced by patients’ baseline condition, surgeons’ judgment, and radiographic findings; therefore, selection bias could not be completely avoided. Although the measured baseline characteristics, including age, sex, bone mineral density, and preoperative local Cobb angle, were comparable between the two groups, treatment decisions may still have been affected by factors not fully captured in the present dataset. Specifically, parameters such as pre-operative fracture mobility or dynamic instability observed on flexion-extension radiographs, the systemic frailty status of elderly patients, potential differences in the distribution of nonunion segments between the thoracic and lumbar vertebrae, and the presence of pre-existing old compression fractures or spinal deformities in adjacent vertebrae were not systematically quantified. These unmeasured baseline variations may partially explain the differences in intermediate-term pain and radiographic outcomes between the PKP and SSF+VP groups. Second, although all included patients had complete follow-up data, the mean follow-up duration was approximately 14 months. Therefore, the present findings should be interpreted as intermediate term rather than long-term outcomes. Longer prospective follow-up is required to determine whether the observed differences in pain relief and Cobb angle maintenance persist over time. Third, the spatial distribution pattern of bone cement was primarily assessed using postoperative two-dimensional anteroposterior and lateral X-rays, rather than standardized three-dimensional CT reconstructions. Although this approach ensured consistency in the evaluation of the entire retrospective study cohort, conventional X-rays may have lower sensitivity than CT in identifying subtle bone cement intermingling, trace bone cement leakage, or complex three-dimensional bone cement distribution within the vertebral body. Future prospective studies using standardized postoperative CT reconstructions are needed to more accurately characterize bone cement distribution patterns and their relationship to clinical outcomes. Fourth, the sample size decreased after subgroup analysis, particularly in the mild-to-moderate kyphosis subgroup, which may have affected the stability of the effect estimates. In addition, residual mild pain was defined as a final follow-up VAS of ≥2. This criterion was based on the pain distribution in the PKP group and the need for clinical interpretation in the present study. Although it can reflect residual mild pain, it remains a study-specific operational definition. Due to the overall low pain scores at the final follow-up, a sensitivity analysis using a severe pain threshold (e.g., VAS ≥4) could not be reliably performed due to data sparsity. Therefore, the ROC-derived preoperative Cobb angle threshold should be interpreted as an exploratory risk-stratification tool rather than an absolute surgical indication. Bootstrap analysis served as an internal validation method and could assess the robustness of the findings within the current sample, but it cannot replace external validation. Further large-sample, multicenter, prospective studies are needed to validate the specific risk threshold of the preoperative Cobb angle and its applicability in surgical decision-making.

## 5 Conclusion

This study demonstrated that both PKP and SSF+VP can effectively improve pain and functional disability in patients with stage III KD without neurological deficits. PKP has the advantages of less surgical trauma and faster early recovery. However, it showed relatively limited performance in maintaining Cobb angle correction and in intermediate-term pain outcomes among patients with severe preoperative kyphosis. In contrast, SSF+VP showed greater advantages in maintaining kyphosis correction and achieving more favorable intermediate-term pain outcomes. In the PKP group, Cobb angle loss was significantly correlated with final follow-up VAS, suggesting that insufficient maintenance of kyphosis correction may be associated with residual mild pain. Meanwhile, the preoperative local Cobb angle may serve as an important radiographic indicator for predicting mild residual pain after PKP. After stratification based on the optimal preoperative Cobb angle cut-off value of 25.95°, SSF+VP showed a more stable advantage over PKP in final follow-up pain outcomes among patients with severe kyphosis. Therefore, for patients with stage III KD without neurological deficits and a larger preoperative Cobb angle, SSF+VP may represent a more appropriate surgical option. Risk stratification based on the preoperative Cobb angle may help guide individualized surgical decision-making in this patient population.

## Data Availability

The raw data supporting the conclusions of this article will be made available by the authors, without undue reservation.
